# Supplementary material for: Association between oral microbiome and seven types of cancers in East Asian population: a two-sample Mendelian randomization analysis
Source: Front Mol Biosci. 2023 Nov 21;10:1327893. doi: 10.3389/fmolb.2023.1327893 (PMC10702768; doi:10.3389/fmolb.2023.1327893)
Supplement: Supplementary file 1 [file DataSheet1.ZIP › Supplementary Materials/MR plots for tongue/tongue═╝/Ovarian cancer/pheno.1252_to_ovarian cancer_forest.pdf]

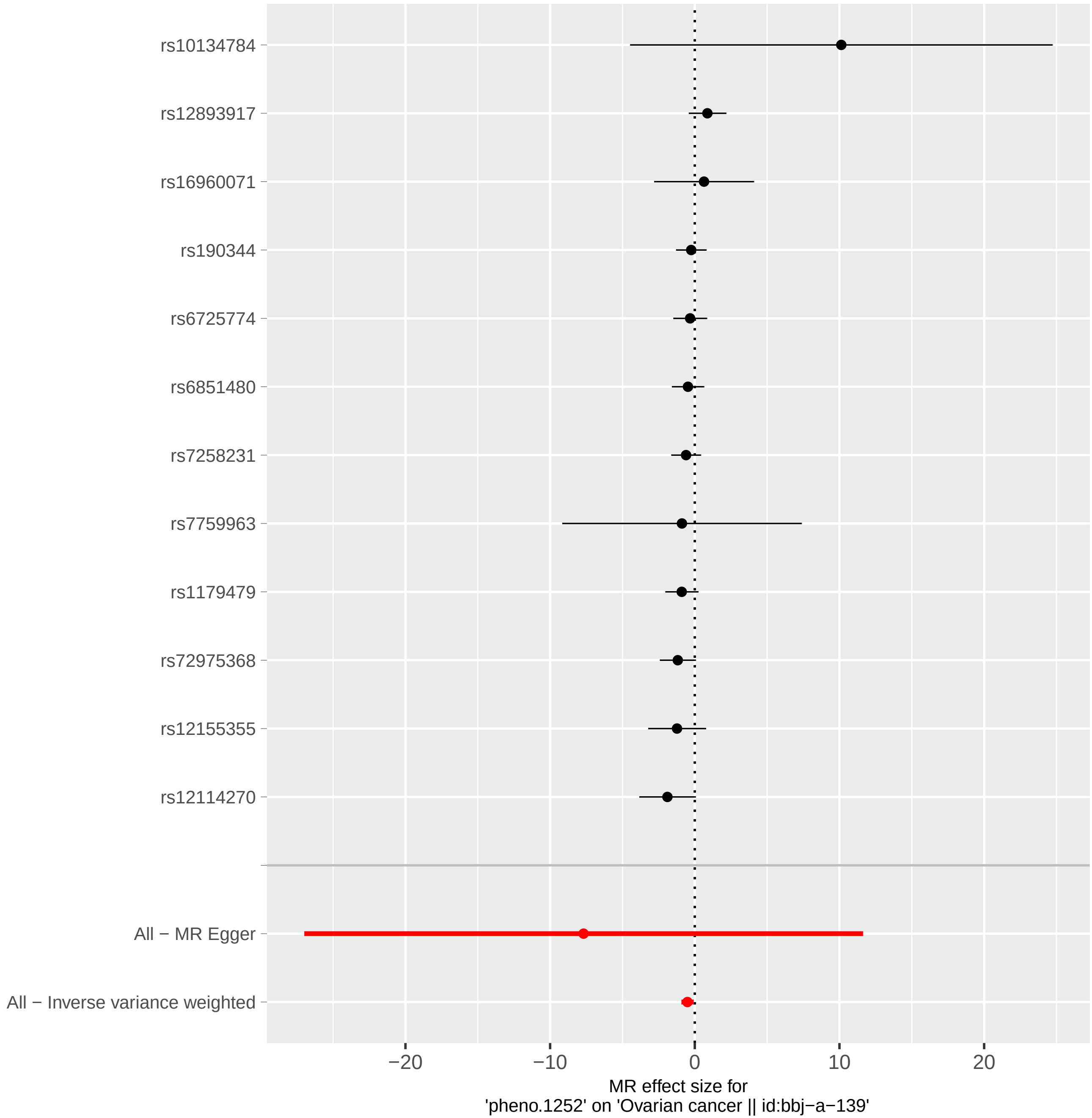
rs10134784
rs12893917
rs16960071
rs190344
rs6725774
rs6851480
rs7258231
rs7759963
rs1179479
rs72975368
rs12155355
rs12114270
All − MR Egger
All − Inverse variance weighted
−20
−10
0
10
20
MR effect size for
'pheno.1252' on 'Ovarian cancer || id:bbj−a−139'
